# Bacterial mimetics of endocrine secretory granules as immobilized *in vivo* depots for functional protein drugs

**DOI:** 10.1038/srep35765

**Published:** 2016-10-24

**Authors:** María Virtudes Céspedes, Yolanda Fernández, Ugutz Unzueta, Rosa Mendoza, Joaquin Seras-Franzoso, Alejando Sánchez-Chardi, Patricia Álamo, Verónica Toledo-Rubio, Neus Ferrer-Miralles, Esther Vázquez, Simó Schwartz, Ibane Abasolo, José Luis Corchero, Ramon Mangues, Antonio Villaverde

**Affiliations:** 1 Institut d’Investigacions Biomèdiques Sant Pau and Josep Carreras Research Institute, Hospital de la Santa Creu i Sant Pau, 08025 Barcelona, Spain; 2 CIBER de Bioingeniería, Biomateriales y Nanomedicina (CIBER-BBN), Spain; 3 CIBBIM-Nanomedicine, Hospital Vall d’Hebron, Universitat Autònoma de Barcelona, 08035, Barcelona, Spain; 4 Institut de Biotecnologia i de Biomedicina, Universitat Autònoma de Barcelona, Bellaterra, 08193 Barcelona, Spain; 5 Servei de Microscòpia, Universitat Autònoma de Barcelona, Bellaterra, 08193 Barcelona, Spain; 6 Departament de Genètica i de Microbiologia, Universitat Autònoma de Barcelona, Bellaterra, 08193 Barcelona, Spain

## Abstract

In the human endocrine system many protein hormones including urotensin, glucagon, obestatin, bombesin and secretin, among others, are supplied from amyloidal secretory granules. These granules form part of the so called functional amyloids, which within the whole aggregome appear to be more abundant than formerly believed. Bacterial inclusion bodies (IBs) are non-toxic, nanostructured functional amyloids whose biological fabrication can be tailored to render materials with defined biophysical properties. Since under physiological conditions they steadily release their building block protein in a soluble and functional form, IBs are considered as mimetics of endocrine secretory granules. We have explored here if the *in vivo* implantation of functional IBs in a given tissue would represent a stable local source of functional protein. Upon intratumoral injection of bacterial IBs formed by a potent protein ligand of CXCR4 we have observed high stability and prevalence of the material in absence of toxicity, accompanied by apoptosis of CXCR4^+^ cells and tumor ablation. Then, the local immobilization of bacterial amyloids formed by therapeutic proteins in tumors or other tissues might represent a promising strategy for a sustained local delivery of protein drugs by mimicking the functional amyloidal architecture of the mammals’ endocrine system.

As gene therapy is not yet of routine application^1^, deficiencies in endogenous proteins must be treated by the administration of externally fabricated proteins for protein replacement or as effective drugs^2^,^3^, including antibodies, hormones or enzymes that exhibit a desired effect based on signalling, receptor blocking or substrate processing^4^. These drugs are delivered by systemic administration, but adverse immune reactions and the high cost of treatments linked to the high doses required pose severe obstacles to this common approach. These constraints are exemplified by the treatment of lysosomal disorders, such as Fabry disease. In the treatment of this condition, 1 mg/kg (Fabrazyme^®^) or 0.2 mg/kg (Replagal^®^) of protein are administered every two weeks to the patient, with a yearly cost depending on the patient weight but usually representing >200.000 €/year/patient^5^. This is also commonly associated to undesired immune reactions that minimize the therapeutic effects of the protein drug. Moreover, for systemically administered drugs, only a small portion of the active compound is expected to reach the target^6^.

In this context, there is a growing interest in the development of new materials as controllable systems for sustained protein release. Ideally, the resulting hybrid materials (scaffold plus functional protein drug) could be immobilized in a given tissue as drug depots for long term local supply of the embedded protein. Then, hydrogels, polymers, and other porous materials and biomaterials are being explored as scaffolds for drug inclusion *in vitro* and further slow release *in vivo*^7^,^8^,^9^,^10^,^11^,^12^,^13^,^14^,^15^,^16^. As in other nanomedical applications, toxicity of the scaffold material is a relevant matter of concern, and the long term stability and interactivity of the scaffold structure itself with body components are critical issues. Then, the use of homogenous delivery platforms in which the protein drug itself is also the immobilizing scaffold would be highly convenient to skip the use of potentially toxic, xenobiotic carrier platforms. In this context, the endocrine system offers some important lessons about sustained protein release from chemically homogenous structures. Many secretory cell types in the human body accumulate functional proteins for long-term storage, in the form of compact protein clusters^17^,^18^. These act as secretory granules that are formed by self-assembling polypeptides with a high extent of internal molecular organization. Such protein reservoirs show an amyloidal nature rich in cross-β sheet contacts that provide stable and robust scaffolds to retain protein as *in vivo* depots. As a response to appropriate stimuli, these functional amyloids release monomeric hormone molecules in a functional form^19^,^20^,^21^,^22^ in a poorly investigated process probably assisted by molecular chaperones^17^.

On the other hand, IBs are cross-β sheet rich, functional amyloids formed in recombinant bacteria^23^ observed as mimetics of the above mentioned secretory granules^24^,^25^,^26^. They combine amyloidal and non-amyloidal protein forms in organized sub-micron particles, in which the amyloidal skeleton offer mechanical stability and retains active, properly folded protein forms through stereospecific interactions^8^,^24^. Any therapeutically valuable protein produced in bacteria can be packaged as IBs^27^, and in addition, bacterial IBs show a high intrinsic and spontaneous penetrability into mammalian cells where they deliver, assisted by molecular chaperones, the functional protein fraction embedded into the amyloidal scaffold^28^. Noting the structural and functional analogies between secretory granules and IBs, the bacterial products would potentially be ideal homogenous materials for sustained protein delivery *in vivo* through bioinspired therapeutic approaches. In this context, we have explored here the potential of IBs formed by a model protein with antitumoral activity, in inducing apoptosis upon prolonged local exposition to target tissues.

## Results and Discussion

**Figure 1:**
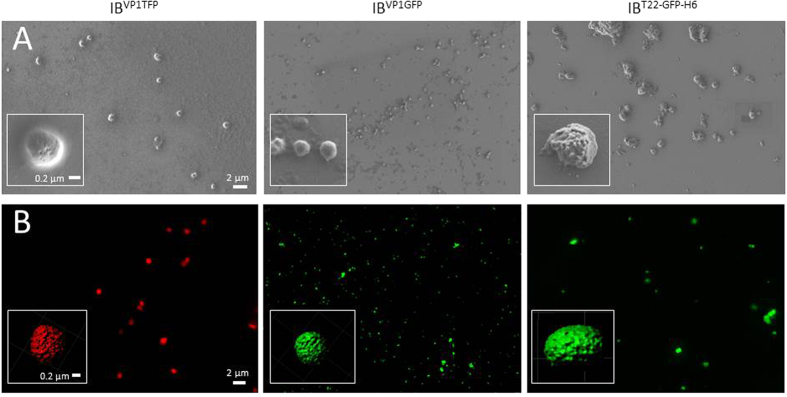
Morphometric analysis of amyloid materials at structural and ultrastructural levels. (**A**) Representative FESEM overviews and details (inboxes) of the amyloidal particulate materials used for immobilization, which occurred as rather homogenous populations. Size range of IB^VP1TFP^ was 400–500 nm, 200–300 nm for IB^VP1GFP^ and 500–600 nm for IB^T22-GFP-H6^ (n > 200). Size bars are common in all images and all inboxes as magnifications are equivalent. (**B**) Broad field confocal images of the materials and 3D IMARIS reconstructions of representative particles (inboxes). Particle sizes here might be largely overestimated due to fluorescence emission.

**Figure 2:**
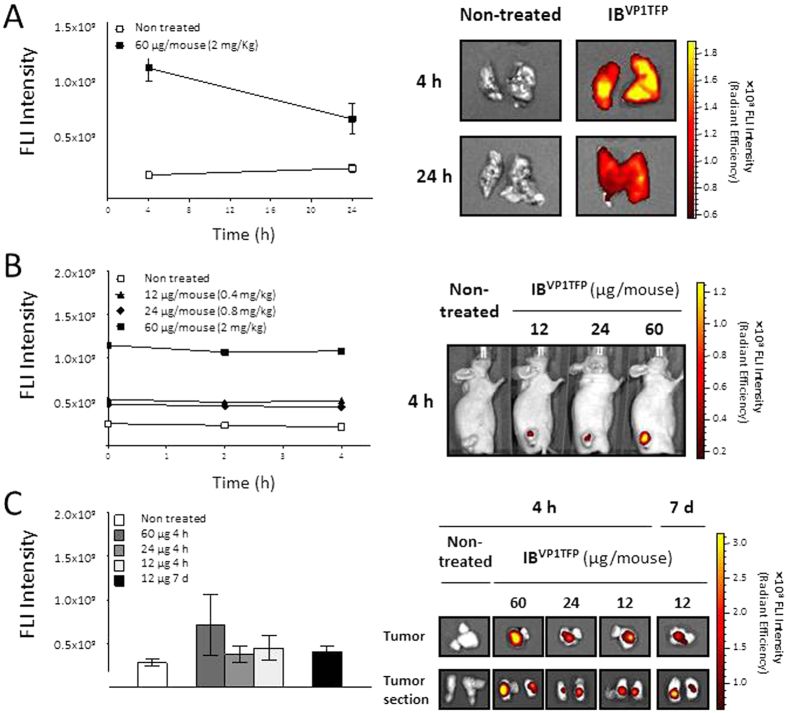
Whole-body biodistribution of fluorescent amyloids upon intravenous or intratumoral administration in a HT-29 colorectal cancer model. (**A**) *Ex vivo* lung fluorescence imaging (FLI) at 4 and 24 h post administration of IB^VP1TFP^ protein particles at 60 μg/mouse by intravenous administration route. *Ex vivo* lung-accumulation of the material was quantified by measuring fluorescent intensity (*left plot*), while representative *ex vivo* fluorescent images of the excised lungs are shown (*right panel*). (**B**) Non-invasive monitoring of IB^VP1TFP^ tumor-accumulations a long time after intratumoral administration at 12, 24 and 60 μg/mouse of IB^VP1TFP^. *In vivo* tumor-accumulation of the material was quantified (*left plot*), and representative *in vivo* fluorescent image of tumor-accumulation at 24 h post administration are shown (*right panel*). (**C**) *Ex vivo* tumor FLI at 4 h after intratumoral administration at 12, 24 and or 60 μg/mouse, and 7 days after intratumoral administration of 12 μg/mouse of the material. *Ex vivo* tumor-accumulation of IB^VP1TFP^ was quantified (*left plot*), and representative *ex vivo* fluorescent images of the excised tumor (whole- and sectioned-tumor) are shown (*right panel*). In all cases, the total tissue-accumulations were quantified by measurements of fluorescent intensity expressed in Radiant Efficiency, MEAN ± SEM. Pseudocolor scale bars were consistent for all images in order to show relative changes for each corresponding images.

Bacterial IBs are regular shaped soft materials that show a moderate polydispersion in size (Fig. 1)^29^,^30^. Their sticky nature^31^ would prevent them from being used in systemic delivery (as aggregation in lungs would be anticipated) while it would instead favour their retention in tissues if administered locally by injection. These predictions were confirmed by intravenous (iv) tail vein administration of suspended fluorescent IBs formed by VP1TFP (IB^VP1TFP^) (as model fluorescent materials suitable to easy monitoring) in subcutaneous (sc) tumor-bearing mice. The *ex vivo* fluorescent imaging (FLI) at early (a few hours) and long-time (1 day) post administration demonstrated that IB^VP1TFP^ got first-pass retained into lung capillaries upon systemic delivery (Fig. 2A), being an indicative of aggregation. Moreover, accumulation of IB^VP1TFP^ into tumor, liver, spleen, kidney, stomach, intestine, heart and skin tissues were not detected at any of the time points studied (4 and 24 h, data not shown). While these data precluded further consideration of IBs as circulating protein drug carrier materials, it was in agreement with possibilities for retention once locally implanted. Solid tumors are highly vascularized tissues, and a poorly stable material would be then easily lost by clearance through the blood stream. In contrast, mechanically stable and firmly attached entities should be predicted as long lasting. Thus, to check the potential of the material as protein depots, IB^VP1TFP^ were intratumorally injected in mice harboring sc tumors of human colon cancer cells. Interestingly, the fluorescent material accumulated in tumor in a dose-dependent way, and the fluorescent light emission signal was stable and steadily localized in the first hours post implantation at each corresponding given dose *in vivo* (Fig. 2B). Moreover, *ex vivo* FLI of tumors 7 days post local IB^VP1TFP^ implantation still showed clusters of the material in the local injection site (Fig. 2C), with steady fluorescence intensities along time for the dose of 12 μg *per* mouse. Even, this fluorescence emission was still within the order of magnitude of the light produced by IB^VP1TFP^ *in vitro* (Supplementary Figure 1). Since IB^VP1TFP^ signal was not found in other tissues (data not shown) no important fractions of the material appeared to have migrated, at least as major detectable clusters. Importantly, no signs of systemic toxicity were observed such as body weight loss or changes in physical appearance or behavioural patterns (data not shown).

These observations, favourable regarding *in vivo* stability of the material even in irrigated tissues, prompted us to explore further the hypothesis of IBs as functional mimetics of secretory granules. For that, we evaluated the availability of the functional IB protein fraction to the cells in the vicinity of the reservoir implantation site. T22 is a peptidic ligand and a CXCR4 antagonist that acts extracellularly by competing with SDF-1α for binding to this membrane receptor^32^. High doses of this peptide trigger the inhibition of signal transduction downstream of CXCR4 leading to proliferative block, apoptotic induction and tumor growth inhibition in CXCR4-overexpressing breast^33^ and brain^34^ cancer models. The protein fusion T22-GFP-H6 was produced in bacteria in form of fluorescent, T22-containing IBs (IB^T22-GFP-H6^) for testing the biological effects of the material when implanted in CXCR4^+^ colorectal cancer models. The coincidence in the same macromolecule of T22 as effector moiety and of GFP as tracking agent renders an unusually convenient system to analyse cell responses with an immediate evidence of protein activity and localization.

**Figure 3:**
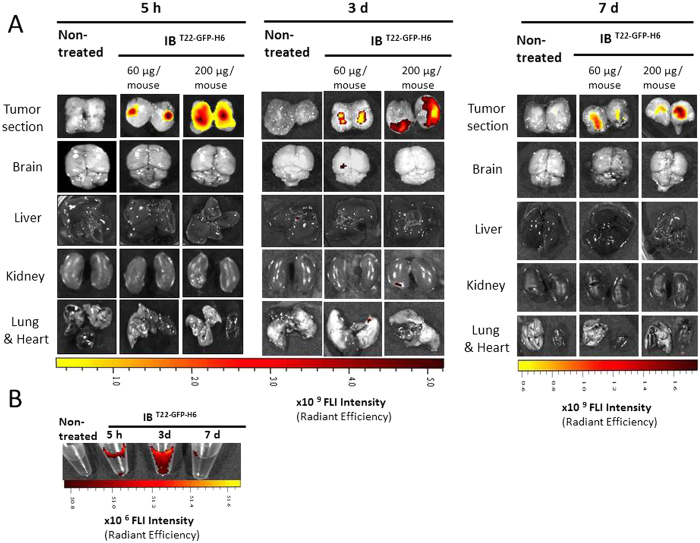
Whole-body biodistribution of functional fluorescent amyloids in CXCR4^+^ colorectal murine model. (**A**) Representative *ex vivo* fluorescent images of the excised tumor and organs (brain, liver, kidney and lung and heart) remaining in tumor tissue at 5 h, and 3 or 7 days post administration of IB^T22-GFP-H6^ protein particles at 60 μg/mouse or 200 μg/mouse using the intratumoral route. (**B**) Fluorescence emitted by soluble protein species in plasma, which were released by IB^T22-GFP-H6^ tumor deposits to the bloodstream at 5 h, and 3 or 7 days after injection of a 200 μg/mouse intratumoral dose. Pseudocolor scale bars were consistent among images in order to show relative changes when being compared.

**Figure 4:**
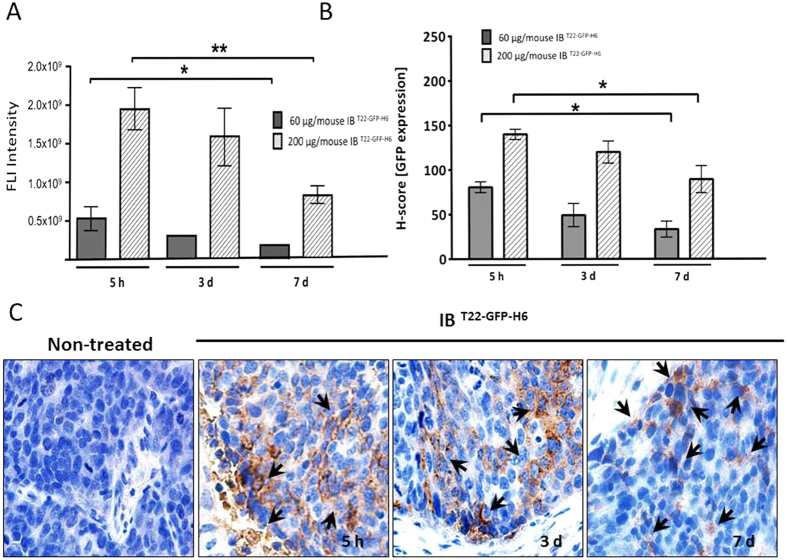
Fluorescent emission by IB^T22-GFP-H6^ deposits remaining in CXCR4^+^ colorectal tumors after their administration. (**A**) Total IB^T22-GFP-H6^ protein deposits were quantified measuring fluorescent intensity at 5h, 3 and 7 days after intratumoral injection of 200 μg/mouse. Data were expressed in Radiant Efficiency. (**B**) Quantitation of total IB^T22-GFP-H6^ protein deposits plus released soluble proteins in tumor tissue calculated as an H-score for anti-GFP immunostaining (brown colour) at 5 h, and 3 or 7 days post administration. (**C**) Representative microphotographs of GFP inmunohistochemistry in IB^T22-GFP-H6^ treated tumors at 5 h, and 3 or 7 days. Note the higher intensity of GFP staining in some tumor areas at 5 h and 3 days (black arrows) and the higher dispersion of protein distribution observed inside the tumor at day 7 post-injection. Quantitative data were expressed as mean ± SE *,**Statistically significant at p < 0.05 or p < 0.01, respectively.

Upon injection in sc tumors, local fluorescence of IB^T22-GFP-H6^ was clearly dose-dependent and largely stable along time (Fig. 3A). About 75 % of the initial fluorescence emission was still linked to the material in tumor 3 days after local injection and decreased only till 40 % in 7 days postinjection (Fig. 4). No toxic side effects were detected in the animals as indicated by lack of body weight loss or clinical signs (not shown). Again, no significant fluorescence levels were observed in analysed organs other than primary tumor (Fig. 3A). Interestingly, the distribution of the IB through the tumor tissue increased with the dose of injected IBs, clearly covering a wider area and indicating a significant level of diffusion of the material and a residual circulating fraction, probably in soluble form since no lung aggregation was observed (Fig. 3B and Table 1). Fluorescent signals were correlated with anti-GFP immunostaining of tumor (Fig. 4B,C) and GFP was detected both in the extracellular matrix and inside tumor cells at any of the time points studied: 5 h, 3 and 7 days (Fig. 4C).

**Figure 5:**
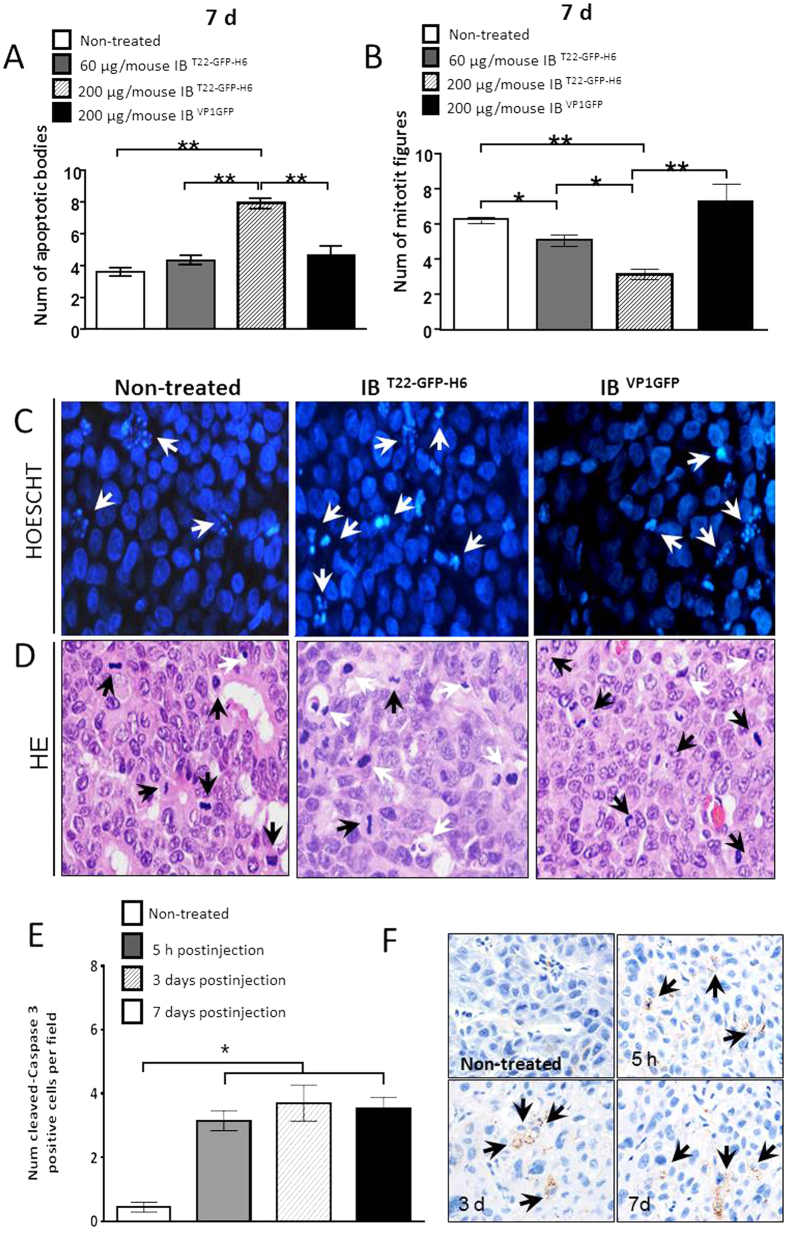
Apoptotic and mitotic index recorded in CXCR4^+^ tumors. Quantitation of apoptotic figures detected by nuclear condensation or nuclear fragmentation after Hoescht staining (**A)** or mitotic figures (**B**) after Hematoxylin-eosin staining in SP5 tumors at day 7 post intratumoral administration of IB^T22-GFP-H6^ protein particles at 60 or 200 μg/mouse. (**C,D**) Representative microphotographs of apoptotic figures by Hoescht ((**C)**, white arrows) or HE ((**D)**, white arrows) staining or mitotic figures ((**D)**, black arrows) by HE staining after local intratumoral injection of 200 μg/mouse dose of targeted IB^T22-GFP-H6^ or non-targeted IB^VP1GFP^ (x 400 magnification). (**E**) Quantitation of the number of positive cells displaying cleaved (active) caspase-3 immunostaining in tumors at 5 h, and 3 or 7 days after intratumoral administration of 200 IB^T22-GFP-H6^ compared to non-treated tumors. (**F**) Representative microphotographs of active caspase-3 positive cells (brown stained tumor cells; black arrows) at the studied time point. Data expressed as mean ± SE *,**Statistically significant at p < 0.05 or p < 0.01, respectively.

When exploring the biological impact of the IB-contained effector T22 on surrounding tissues, we observed a dramatic occurrence of apoptotic events in the tumor tissue (Fig. 5A,C), that was not observed for the control, closely related T22-devoid IB^VP1GFP^. The number of apoptotic bodies was unperceived 5 h after injection but they rose at 3 days and 7 days, in parallel with a significant drop in the number of mitotic cells, not observed when implanting control, non-functional IBs (Fig. 5B,D). The coincidence of inhibited cell proliferation and apoptotic induction was indicative of the fusion protein being available to the surrounding tissues in the course of prolonged time periods. This apoptotic induction was associated with sustained caspase-3 activation along time (Fig. 5E,F).

**Figure 6:**
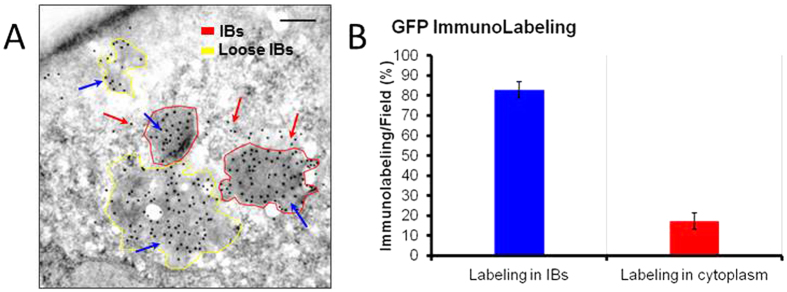
Protein release from IBs. (**A**) Immunogold labeling of GFP in HeLa cells after 24 h of exposure to VP1-GFP-H6 IBs. Blue arrows indicate labeling in the IB particle while red arrows indicates labeling of released protein to the cell cytoplasm. Highly electrodense IB particles with standard morphometries are shadowed in red while those with loose morphologies and showing lower electrodensity, in yellow. The scale bar represents 200 nm. (**B**) Quantification of IB-attached and IB-free immunolabeling signals at this incubation time.

Around 20 % of the IB protein is organized as proteinase K-resistant amyloid fibers^35^,^36^, in a pattern similar to that of protein hormones in the human secretory system. Such amyloidal network ensures a stable fibrous scaffold that confers mechanical stability and high porosity to IBs enabling them to be used as functional biomaterials^26^,^37^. The remaining IB protein fraction occurs embedded within this network probably by sterospecific interactions^35^,^38^, as native-like rich protein versions that are releasable upon internalization by mammalian cells^39^. In fact, upon *in vitro* incubation with physiological buffer, an immediate but time-limited amount of IB protein (about 5 %) is released from IBs in the active form^40^. Such protein loss is probably occurring through an equilibrium between soluble and aggregated protein species, with a kinetics controlled by an aggregation/dissociation constant. However, the fraction of released functional protein appears to be higher upon internalization into mammalian cells, what might be linked to the suggested involvement of cell chaperones in the disintegration of exogenous IBs^41^,^42^,^43^,^44^ and not subjected to physicochemical equilibrium constants. To confirm such protein release and its time-prolonged nature we determined, by GFP immunostaining, the amount of IB protein still associated to engulfed IBs and the IB protein fraction free from the bulk material, in the cell cytoplasm. As observed (Fig. 6A,B), more than 20% of IB protein is found free in the cell cytoplasm, 24 h after exposure of cultured mammalian cells to IB^VP1GFP^. Interestingly, some IBs show their natural electrodense nature under transmission electron microscopy (Fig. 6A, red borders) while others are much loser and less dense (Fig. 6A, yellow borders). These images are compatible with those observed upon proteinase K treatment of isolated IBs, in which an important fraction of the protein is degraded and the amyloidal skeleton, free from functional protein, remains^36^. This amyloidal network represents about 20 % of the total recombinant protein in IBs, but this percentage, as well as the amount of functional protein that can be released from IBs seems to be regulatable by the proper choice of the genetic background of the bacteria where IBs are produced^36^, and by the production conditions, such as temperature and others^45^,^46^,^47^. The potential to obtain IBs with variable amounts of releasable protein and with different global conformational status^48^,^49^ might offer opportunities for a better control of the protein release upon injection, as well as a way to minimize the amount of unfolded protein, what could contribute to undesired immune responses.

In addition to the releasable nature of a fraction of IB protein, the high inherent membrane-activity of bacterial IBs allows efficient adhesion and cell penetration in absence of cytotoxicity, properties that justified the exploration of IBs as both functional topographies in tissue engineering and as protein delivery agents in replacement therapies^24^. In this context, the absence of intrinsic toxicity of bacterial IBs has been widely demonstrated by conventional procedures on IBs formed by diverse proteins with potential biomedical interest, in therapeutic/prophylactic setting ups. This includes cytoskeleton proteins^50^, enzymes^39^, chaperones^39^ and growth factors^42^,^43^,^51^ among others, and very recently, cytokines in a Zebrafish animal model^52^.

Beyond the well-known applications of bacterial IBs as vehicles for functional protein in cell culture and oral administration^53^,^54^,^55^,^56^, we demonstrate here that bacterial IBs act as *in vivo* depots of functional proteins upon their immobilization by local injection. The availability for interaction and signalling of a CXCR4 peptide ligand that was part of an IB-forming fusion protein and the dramatic impact promoting apoptosis in primary tumor fully confirm the successful mimicry of IBs as implantable homogenous materials for sustained protein release. The fact that the tissue volume in which IBs occur is dose-dependent and expands with increasing IB amounts (Figs 2B,C and 3B,C) opens a possibility to regulate the zone of therapeutic impact, what could be also defined by the selection of multiple injection sites, if required.

Finally, the recent identification of IB formation in food-grade bacteria^57^, the development of endotoxin-free *E. coli* strains^58^ and their adaptation to the production of endotoxin-free IBs^59^ further expand the opportunities for the *in vivo* uses of IBs, supporting again the added values of biofabrication *versus* chemical synthesis in the production of functional materials.

## Methods

### Bacterial production of IBs

VP1TFP is a far-red fluorescent protein TurboFP635 fused to an aggregation prone viral peptide (from foot-and-mouth disease virus). VP1GFP is an equivalent protein containing the enhanced GFP, while T22-GFP-H6 contains T22 instead of VP1. IB versions of these proteins (Fig. 1) were produced from encoding plasmid vectors by IPTG-mediated induction in *E. coli* as described^39^,^60^,^61^, purified by a stringent protocol designed to minimize molecular contamination from the producing cells^62^, and strictly tested by the absence of contaminating, full cell bacteria by described procedures^62^. The absence of toxicity of IBs on mammalian cell cultures was regularly determined by conventional MTT assays, resulting in viability values always over 90 % (usually over 95 %; not shown). This is coincident with multiple reports showing the lack of intrinsic cytotoxicity of IB^VP1GFP^ and IB^TF1GFP^ produced by optimized protocols^28^,^39^,^61^,^63^. Protein production and IB purification has been partially performed by the ICTS “NANBIOSIS”, more specifically by the Protein Production Platform of CIBER in Bioengineering, Biomaterials & Nanomedicine (CIBER-BBN)/ IBB, at the UAB SepBioES scientific-technical service.

### Field Emission Scanning Electron Microscopy (FESEM)

The ultrastructural details of size, shape and surface aspect were evaluated at nearly native state with a Field Emission Scanning Electron Microscope (FESEM). Purified IBs were diluted in water and microdrops of 10 μl of each sample were deposited over silicon wafers (Ted Pella, Reading, CA, USA) and air dried. Then, the materials were immediately observed without coating in a FESEM Zeiss Merlin (Zeiss, Oberkochen, Germany), operating at 2 kV and equipped with a high resolution in-lens secondary electron detector.

### *In vivo* experiments

All *in vivo* experiments were performed by the ICTS “NANBIOSIS”, more specifically by the CIBER-BBN’s *in vivo* Experimental Platform of the Functional Validation & Preclinical Research (FVPR) area (http://www.nanbiosis.es/unit/u20-in-vivo-experimental-platform/) and the Nanotoxicology platform of IIB Sant Pau (http://www.nanbiosis.es/portfolio/u18-nanotoxicology-unit/. Animal care was handled in accordance with the Guide for the Care and Use of Laboratory Animals of the involved institutions and all the *in vivo* procedures were approved by the Hospital de Sant Pau and Hospital Vall d’Hebron Animal Ethics Committees and performed according to EC directives. Detailed protocols are given in the Supplementary information.

## Additional Information

**How to cite this article**: Céspedes, M. V. *et al*. Bacterial mimetics of endocrine secretory granules as immobilized *in vivo* depots for functional protein drugs. *Sci. Rep.* **6**, 35765; doi: 10.1038/srep35765 (2016).

## Supplementary Material

Supplementary Information

## Figures and Tables

**Table 1:** Kinetics of IBT22-GFP-H6 protein depots remaining in tumor and soluble species released to plasma, and their antitumor effect in tumor tissue.

Parameter	Time			
	0 hours	5 hours	3 days	7 days
IBs in tumor (μg)	200	184.5	155.3	87.9
IHC GFP H-score	N.d	140 ± 5	120 ± 12	89 ± 15
Cleaved-Caspase 3	N.d	3.2 ± 0.3	3.7 ± 0.6	3.5 ± 0.4
Apoptosis	N.d	3.7 ± 0.2	5.9 ± 1	8.0 ± 0.2
Mitosis	N.d	4.9 ± 0.3	3.9 ± 0.7	3.1 ± 0.1
Plasma (μg/ml)	0	2.9	16.4	0

Samples were analyzed at 0 h, 5 h, 3 and 7 days after intratumoral injection at a 200 μg/mouse dose.

IHC GFP Score: Immunohistochemical quantitation of proteins containing GFP in tumor tissue N.d.: not done.
