# Supplementary material for: Bacterial mimetics of endocrine secretory granules as immobilized in vivo depots for functional protein drugs
Source: Sci Rep. 2016 Oct 24;6:35765. doi: 10.1038/srep35765 (PMC5075894; doi:10.1038/srep35765)
Supplement: Supplementary Information [file srep35765-s1.doc]

Bacterial mimetics of endocrine secretory granules as immobilized *in vivo* depots for functional protein drugs

María Virtudes Céspedes ^1,2 Ϯ^, Yolanda Fernández ^2,3 Ϯ^, Ugutz Unzueta ^1,2^, Rosa Mendoza ^2,4^, Joaquin Seras-Franzoso ^2,3^, Alejando Sánchez-Chardi ^5^, Patricia Álamo ^1,2^, Verónica Toledo-Rubio ^2,4,6 £^, Neus Ferrer-Miralles ^2,4,6^, Esther Vázquez ^1,2^ Simó Schwartz Jr.^2,3^, Ibane Abasolo ^2,3^, José Luis Corchero ^2,4,6*^, Ramon Mangues ^1,2^, Antonio Villaverde ^2,4,6*^

^1^ Institut d'Investigacions Biomèdiques Sant Pau and Josep Carreras Research Institute, Hospital de la Santa Creu i Sant Pau, 08025 Barcelona, Spain.

^2^ CIBER de Bioingeniería, Biomateriales y Nanomedicina (CIBER-BBN), Spain

^3^ Cibbim-Nanomedicine, Hospital Vall d'Hebron, Universitat Autònoma de Barcelona, 08035, Barcelona, Spain

^4^ Institut de Biotecnologia i de Biomedicina, Universitat Autònoma de Barcelona, Bellaterra, 08193 Barcelona, Spain

^5^ Servei de Microscòpia, Universitat Autònoma de Barcelona, Bellaterra, 08193 Barcelona, Spain

^6^ Departament de Genètica i de Microbiologia, Universitat Autònoma de Barcelona, Bellaterra, 08193 Barcelona, Spain

^Ϯ^ Equally contributed

^£^ Present address: Bioingenium S.L. Parc Científic de Barcelona, Edifici Hèlix, Baldiri Reixac 15-21, 08028 Barcelona, Spain

^*^Corresponding authors: JLC, jlcorchero@ciber-bbn.es; AV, [Antoni.Villaverde@uab.es](mailto:Antoni.Villaverde@uab.es)

**Supplementary information**

**Subcutaneous colorectal cancer mouse models**

Female athymic nude mice (non functional T cells and functional B and NK cells) were kept in pathogen-free conditions and used at 6 weeks of age.

HT-29 colorectal cancer (CRC) model. Human CRC HT-29 cells (1×10^6^, from the ATCC, Rockville, MD, USA) were injected subcutaneously (sc) on the rear flanks of the mice (Harlan Interfauna Iberica, Barcelona, Spain). Tumor growth was monitored twice a week for 4 weeks by conventional caliper measurements (D×d2/2, where *D* is the major diameter and *d* the minor diameter). Once the tumors reached a median volume of ~200 mm^3^, mice were randomized in the different treatment groups according to their tumor volume, and the biodistribution studies were performed.

SP5 CXCR4+ CRC model. Ten mg of SP5 CXCR4+ tumor tissue from donor animals were obtained and implanted sc in the subcutis of mice (Charles River, L-Abreslle, France). When tumors reached 500 mm^3^ approximately, mice were randomly allocated and biodistribution was performed.

**IBs intravenous administration and intratumoral deposition of amyloids**

Pellets of known amounts of IB^VP1TFP^, IB^T22-GFP-H6^ and IB^VP1GFP^ were resuspended in PBS pH 7.4, at 4 mg/mL. IB^VP1TFP^ were administered to tumor-bearing mice by intravenous administration, with a single dose of 60 µg/mouse (2 mg/kg) or by intratumoral administration at different doses (12, 24 and 60 µg/mouse). IB^T22-GFP-H6^ were administered intratumoraly to anesthetized (100 mg ketamine/ 10 mg xylazine) mice at a single dose of 60 or 200 µg/mouse (n=3). IB^VP1GFP^ were administered at a single dose of 200 µg/mouse (n=2). In all cases, a non-treated tumor-bearing mouse (administered with PBS solution) was included as an autofluorescent control.

***In vivo* and *ex vivo* whole-body biodistribution**

The *in vivo* tumor-accumulation and whole-body biodistribution of the fluorescent IBs was monitored noninvasively by fluorescent imaging (FLI) using the IVIS Spectrum imaging system (PerkinElmer Life Science, Waltham, MA, USA), and images and measurements were acquired and analyzed using the Living Image 4.3 software (PerkinElmer). Animals were anesthetized using 1–3 % isoflurane (Abbott Laboratories, Abbott Park, IL) and imaged. Then, mice were euthanized at 4 and 24 h post administration for the intravenous administration route, and at 0, 2, 4 h and 7 days post administration for the intratumoral administrations of IB^VP1TFP^ and at 5 h and 7 days for the intratumoral administrations of IB^T22-GFP-H6^ and IB^VP1GFP^. Tumors and the major organs such as lungs, brain, liver, spleen, kidneys, stomach, intestine, heart and skin were dissected from mice, weighted and imaged by *ex vivo* FLI of TFP or GFP. Tissues samples were harvested and stored at −80 °C. The light emitted by fluorescent IB^VP1TFP^, IB^T22-GFP-H6^ and IB^VP1GFP^ was detected, digitalized and electronically displayed as a pseudocolor overlay onto a gray scale animal image. Regions of interest from displayed images were drawn manually around the fluorescent signals and quantified as Radiant Efficiency. All the analyses and graphs were performed using Prism 5 software (GraphPad, San Diego, CA, USA). Finally, all organs were collected and fixed with 4 % formaldehyde in phosphate-buffered solution for 24 h. They were then embedded in paraffin for histological analyses and proliferation and apoptotic index evaluation.

**Mouse blood collection and plasma kinetics**

Plasma was obtained from 1ml of blood collected from each mouse by intracardiac puncture. Fluorescence emitted by the obtained plasma was, then, recorded ex-vivo using the IVIS equipment at time 0 h, 5 h, 3 days and 7 days after the administration of a single 200 μg intratumoral dose of IB ^T22-GFP-H6^.

**Histopathology and inmunohistochemistry analyses**

Four-micrometer-thick sections were stained with hematoxylin and eosin (H&E), and a complete histopathological analysis was performed by two independent observers. Tumor slices were also processed to assess proliferation capacity by counting the number of mitotic figures *per* ten high-power fields (magnification x400). The presence of cell death bodies was assayed by H&E and also by Hoescht staining in Triton X-100 (0.5 %) permeabilized sections. After staining the slides with Hoescht 33258 (Sigma-Aldrich Co.) (1:5000 in PBS) for 1 h, rinsed with water, mounted and analyzed under fluorescence microscope (λex=334 nm/λem=465 nm). The number of apoptotic bodies was quantified by recording the number of condensed nuclei per 10 high-power fields (magnification 400x).

The presence and location of the GFP domain of the T22-GFP-H6 protein or the active cleaved-Caspase 3 protein in tissue sections were assessed by immunohistochemistry using the DAKO immunosystem equipment and standard protocols. A primary antibody against GFP (1:100; Santa Cruz Biotechnology, Inc., Santa Cruz, CA) or anti-active caspase 3 antibody (1:300, BD PharMigen, San Diego, CA, USA) were incubated for 25 min to detect GFP presence and location and the level of caspase 3 activation, after incubation with the secondary antibody, in tumor tissues at 5h, 3 and 7 days. The percent of CXCR4-expressing cells in relation to the total cell number and their staining intensity was quantified, scoring each from 0 to 3 (where 3 is the maximal intensity) and multiplying both values to obtain the H-score. The number of stained cells was quantified by counting the number of positive cells per 10 high-power fields (magnification 400x). Representative pictures were taken using Cell^∧^B software (Olympus Soft Imaging v 3.3, Japan). Differences in signals were analyzed by a non-parametric Mann-Whitney test.

**Electron microscopy**

HeLa cells cultured in T75 flasks and exposed to 375 µg of IB^VP1GFP^ for 24 h were treated for Transmission Electron Microscopy and further immunolabelling as described ^1^. Quantitation of immune gold signals was done by using Image J software. Briefly, electrodense Region of Interest (ROI) was defined corresponding to the internalized IBs and antibody, conjugated to gold nanoparticles, inside and outside ROI counted in five independent fields.

**Supplementary Figure 1**


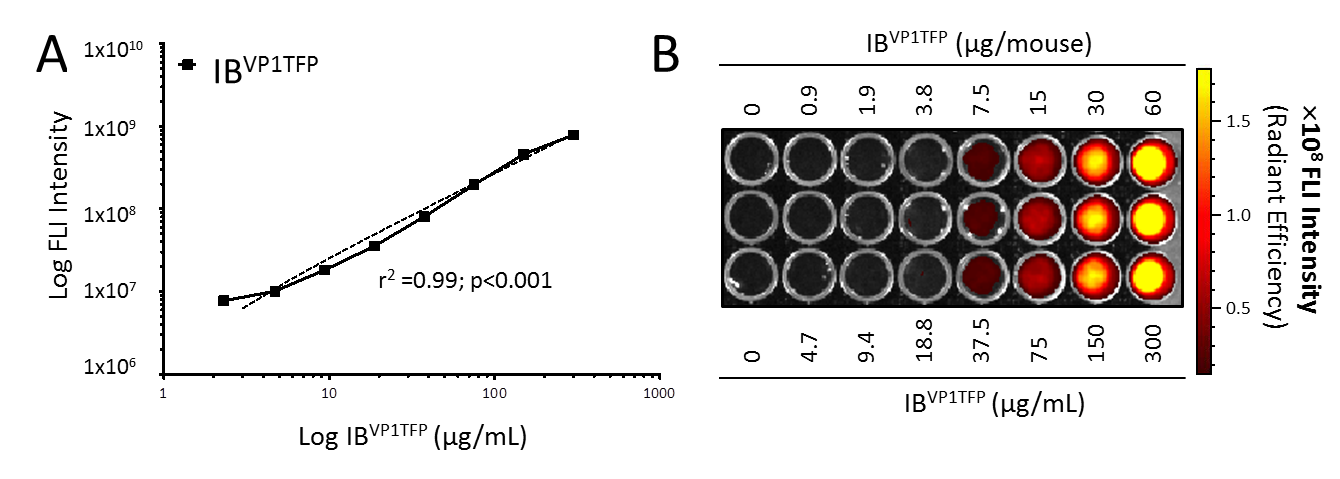


**Supplementary Figure 1: Fluorescent light production of IB^VP1TFP^ *in vitro***. Protein particles were diluted from 60 to 0.05 µg (300 to 0.3 µg/mL) at ½ serial dilutions and added to wells in 96-well black plates. The fluorescent light production of the particles was determined by means of FLI *in vitro* using the IVIS Spectrum imaging system. (**A**) IB^VP1TFP^ fluorescent light production was quantified by Radiant Efficiency and plotted against protein concentration. The dotted line indicates the linear regression fit of the correlation. (**B**) Representative fluorescent image of serial two-fold dilutions of IB^VP1TFP^.


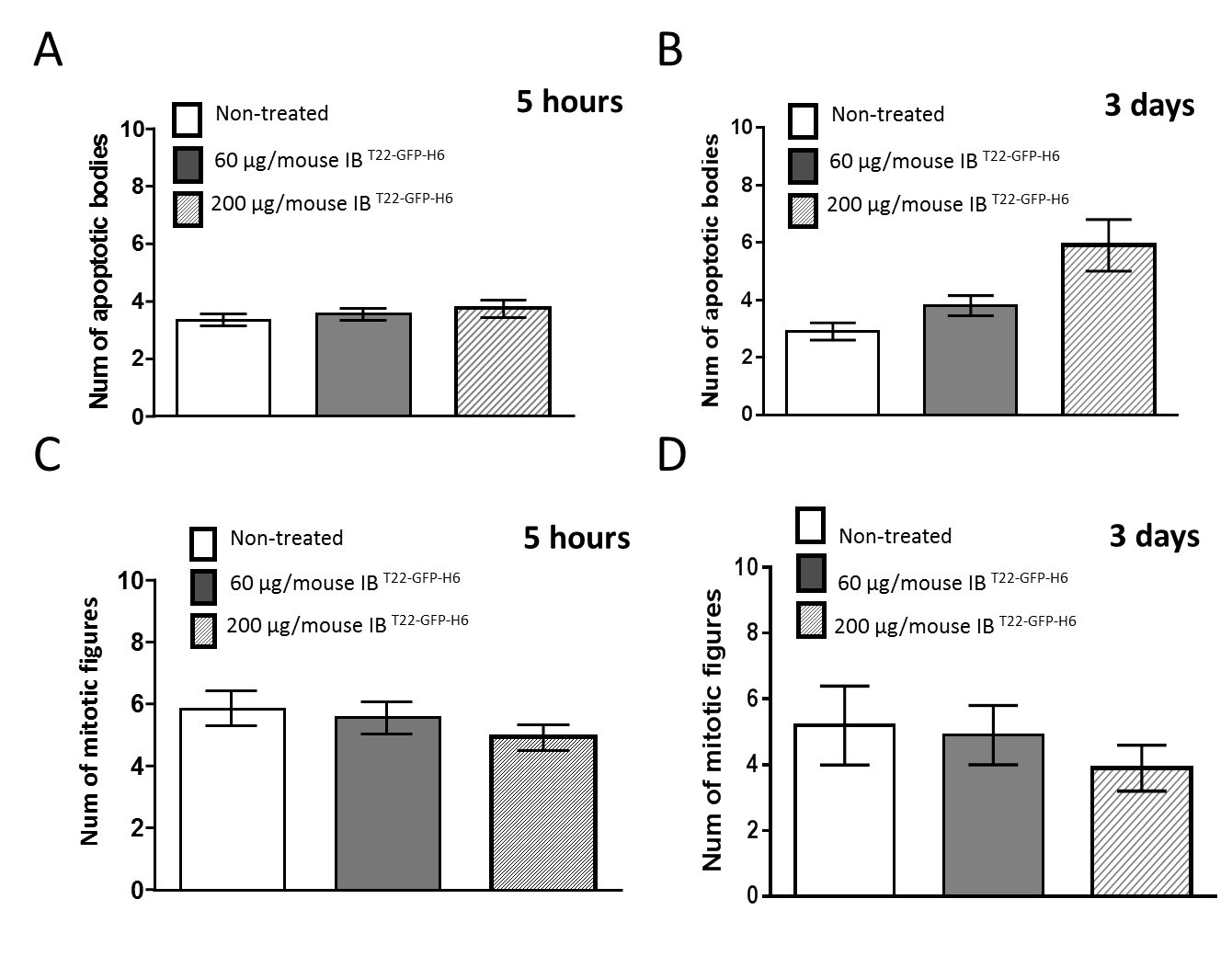


**Supplementary Figure 2: Apoptotic and mitotic index in CXCR4^+^ tumors at 5 hours and 3 days after the intratumoral injection of IB^T22-GFP-H6^.** Quantitation of apoptotic figures detected by nuclear condensation or nuclear fragmentation after Hoescht staining (**A, B**)**,**  or mitotic figures (**C, D**) after Hematoxylin-eosin staining in SP5 tumors at 5 hours and 3 days post intratumoral administration of IB^T22-GFP-H6^ particles at 60 or 200 µg/mouse. Data are expressed as mean±SE .

Reference List

1. Seras-Franzoso,J., Sanchez-Chardi,A., Garcia-Fruitos,E., Vazquez,E., & Villaverde,A. Cellular uptake and intracellular fate of protein releasing bacterial amyloids in mammalian cells. Soft. Matter **12**, 3451-3460 (2016).
